# Anatomical Parameters of tDCS to Modulate the Motor System after Stroke: A Review

**DOI:** 10.3389/fneur.2017.00029

**Published:** 2017-02-09

**Authors:** Stephanie Lefebvre, Sook-Lei Liew

**Affiliations:** ^1^Neural Plasticity and Neurorehabilitation Laboratory, Chan Division of Occupational Science and Occupational Therapy, Division of Biokinesiology and Physical Therapy, Department of Neurology, Stevens Neuroimaging and Informatics Institute, University of Southern California, Los Angeles, CA, USA

**Keywords:** tDCS, stroke, motor recovery, electrode placement, optimal stimulation parameters

## Abstract

Transcranial direct current stimulation (tDCS) is a non-invasive brain stimulation method to modulate the local field potential in neural tissue and consequently, cortical excitability. As tDCS is relatively portable, affordable, and accessible, the applications of tDCS to probe brain–behavior connections have rapidly increased in the last 10 years. One of the most promising applications is the use of tDCS to modulate excitability in the motor cortex after stroke and promote motor recovery. However, the results of clinical studies implementing tDCS to modulate motor excitability have been highly variable, with some studies demonstrating that as many as 50% or more of patients fail to show a response to stimulation. Much effort has therefore been dedicated to understand the sources of variability affecting tDCS efficacy. Possible suspects include the placement of the electrodes, task parameters during stimulation, dosing (current amplitude, duration of stimulation, frequency of stimulation), individual states (e.g., anxiety, motivation, attention), and more. In this review, we first briefly review potential sources of variability specific to stroke motor recovery following tDCS. We then examine how the anatomical variability in tDCS placement [e.g., neural target(s) and montages employed] may alter the neuromodulatory effects that tDCS exerts on the post-stroke motor system.

## Introduction

Stroke is a neurological deficit induced by the interruption of the blood flow to the brain due to either a vessel occlusion or less frequently an intracerebral hemorrhage (1). Both may induce direct damage of brain tissue at the site of the lesion, along with potential for additional damage in the surrounding tissue, and long-range dysfunction through the interruption of structural and functional pathways in the brain. This also leads to a deregulation of cortical excitability (2–4) and abnormal interhemispheric interactions. Stroke may thus induce many neurological deficits and could result in death. According to the World Stroke Organization, one out of six people will suffer from a stroke, making stroke a leading cause of adult long-term disability worldwide (5–7). Importantly, one of the main challenges after stroke is the loss of one’s functional motor abilities. Research suggests that only 12% of stroke survivors achieve complete motor recovery by 6 months after the stroke (8). In addition, older individuals are more vulnerable to stroke and thus the incidence of stroke is expected to continue rising over the next few decades (9, 10). Accordingly, there is a need to find new potential therapeutic tools to enhance post-stroke motor recovery. Rebalancing interhemispheric interactions and/or restoring excitability in the ipsilesional hemisphere is thought to be beneficial for post-stroke motor recovery (11–17). Thus, techniques aimed at restoring functional brain activity are a promising way to enhance neural recovery after injury. Most of the literature on stroke recovery focuses on the recovery of upper limb motor function. Since the neural mechanisms involved in motor recovery of upper versus lower limbs may differ, in this review, we focus only on upper limb motor recovery after stroke.

**Figure 1:**
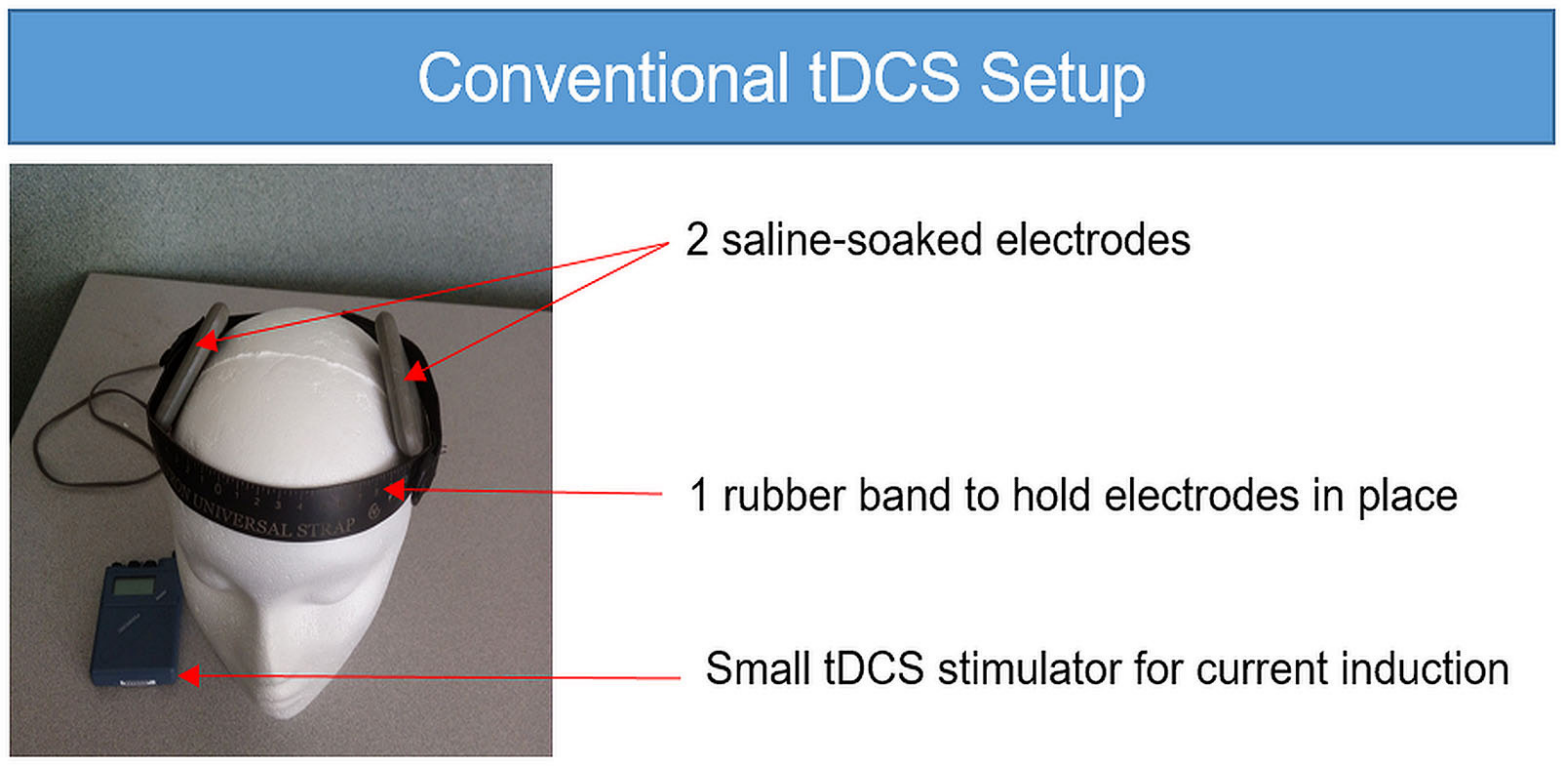
**Conventional transcranial direct current stimulation (tDCS) setup**. The conventional tDCS setup requires a small tDCS stimulator with a 9-V battery, two saline-soaked sponge electrodes and one rubber band to hold the electrodes in place on the head. While there are many options for convention tDCS, the unit shown here is the Chattanooga Iontophoresis device.

**Figure 2:**
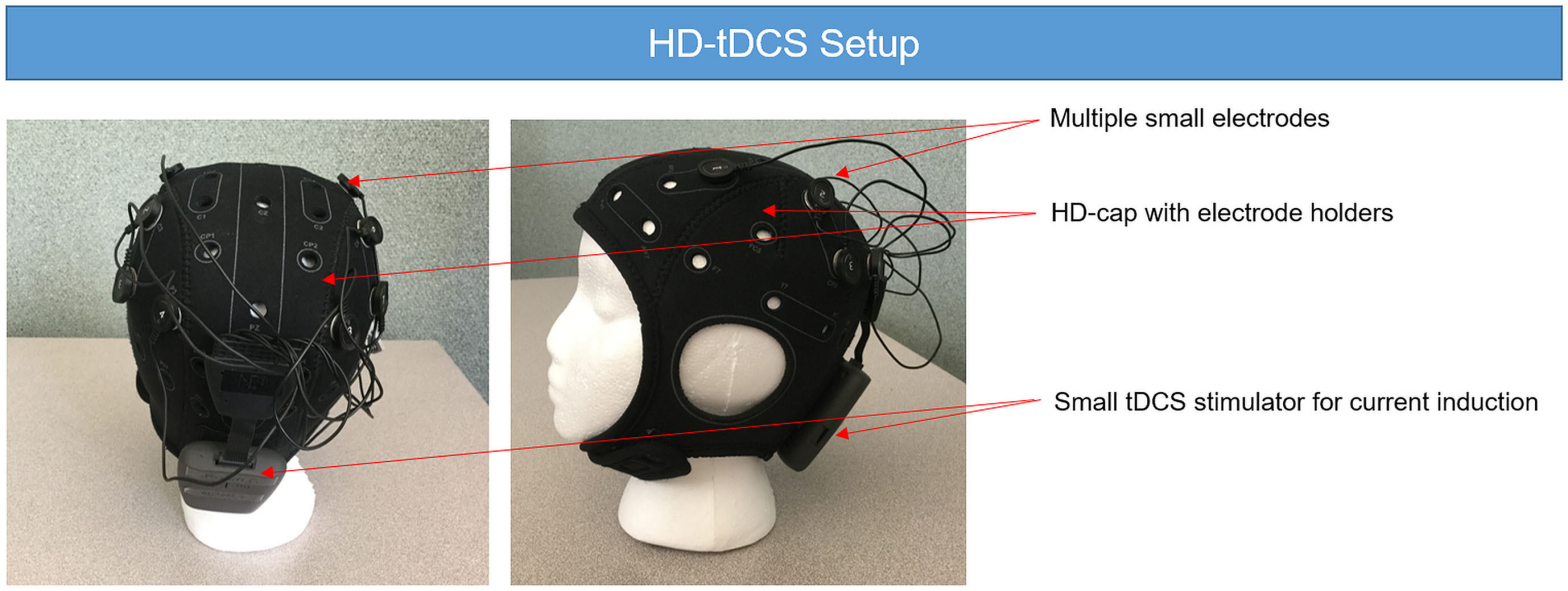
**High-definition tDCS (HD-tDCS) setup**. The HD-tDCS setup requires the use of several small electrodes to build the desire montage. The electrodes are fixed on the HD-cap. The parameters of the stimulation are stored in the small transcranial direct current stimulation (tDCS) stimulator attached at the back of the head and usually modeled using a computer program. While there are several options for HD-tDCS, the unit shown here is the Neuroelectrics Starstim device.

Non-invasive brain stimulation (NIBS) techniques show strong therapeutic potential for post-stroke motor rehabilitation due to their ability to modulate cortical excitability (18–21). In particular, transcranial direct current stimulation (tDCS) has emerged as a viable neurorehabilitation tool due to its limited side-effects (22, 23) and safety [e.g., no known risk of neural damage or induction of seizures, as can be found in other NIBS methods like repetitive transcranial magnetic stimulation (rTMS) (24, 25)]. In addition, tDCS stimulators are commercially available and relatively affordable, on the order of several hundred dollars, and application of tDCS is considered relatively simple. By delivering a low-intensity direct current (between 0.5 and 2 mA) to the scalp *via* two saline-soaked electrodes—an anode and a cathode—tDCS can modulate the transmembrane potential of neurons, modifying cortical excitability and inducing changes in neural plasticity (see Figure 1) (26–30). In addition, recent work has attempted to enhance the spatial resolution of tDCS stimulation, using a new technique called high-definition tDCS (HD-tDCS) (31–34). With this technique, brain regions are more focally targeted using arrays of smaller electrodes arranged on the scalp (Figure 2), using multiple anodes and cathodes (see section on Focal versus Broad Stimulation for a more detailed description). Recently, there has also been increased interest in combining tDCS with imaging methods, such as fMRI or EEG, in order to better understand the local and global effects of tDCS on neural plasticity throughout the brain (35). These methods have all contributed to the growth and interest of tDCS as a viable neuromodulatory method for stroke.

**Figure 3:**
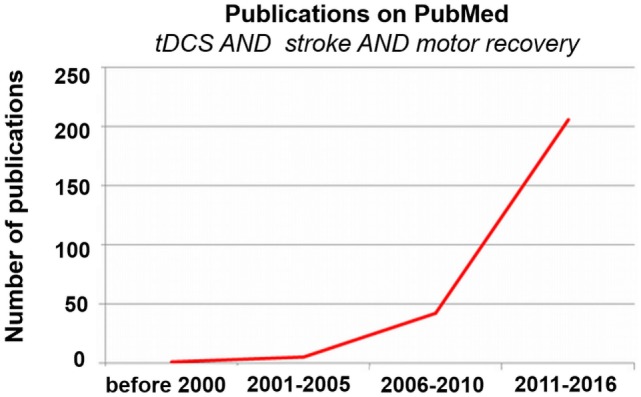
**Publications on transcranial direct current stimulation (tDCS), stroke, and motor recovery**. Exponentially increasing number of publications on tDCS, stroke, and motor recovery *via* PubMed over time.

Accordingly, the use of tDCS to enhance motor recovery after stroke has grown rapidly in the last 10 years (see Figure 3). Despite the increase in research on tDCS for stroke motor recovery, there is much variability in the reliability of this method, with some studies finding that up to 50% or more of patients do not show a change in behavior or cortical excitability following stimulation (36–38). Several stimulation parameters may have an influence on efficacy of tDCS in individuals with stroke, including (1) the placement of the electrodes (e.g., the montage and the neural targets), (2) the intensity of the current, (3) the duration of the stimulation, (4) the timing of the stimulation (e.g., when the stimulation should be applied), (5) the use of a concomitant task (and the nature of the task), (6) the time since stroke, and (7) the stroke lesion size and location, among many other variables. Several provocative and informative reviews provide more insight into these topics (39–43). While little is known overall about the optimal stimulation parameters to enhance motor recovery, work is being done on various aspects of this question. For instance, several researchers are focusing on the optimal tDCS dose (i.e., current intensity, duration, and timing of the stimulation) (44, 45). However, the first question one might ask when designing a tDCS study is not the dose of the stimulation but which brain areas should be targeted and with which kind of stimulation (i.e., excitatory or inhibitory). While there are many conceptual models from which neural targets can be derived to potentially enhance stroke recovery, in the current review, we focus on the motor network, comprised of regions in the primary motor, premotor, supplementary motor, and cerebellar areas, as well as tangential regions, such as the dorsolateral prefrontal cortex (DLPFC). We discuss the potential of targeting each region in relation to stroke recovery. We also touch briefly on the potential reasons for the observed variability in stroke tDCS studies, and then focus more in-depth on the placement of the electrodes.

## Variability and Lack of Reliability of tDCS Studies in Individuals with Stroke

The variable effects of tDCS on motor performance may be due to the lack of standardization of the technical parameters used across sites. More likely, however, it is also due to (1) the heterogeneity of the populations involved in the studies (43) and (2) the variability of the motor paradigms used.

### Heterogeneity of the Involved Populations

The heterogeneity of each study population is a potential source of variability for tDCS effects. However, researchers have had difficulty in finding consistent sources of variability. Studies typically examine the effects of (1) individual biological variability (e.g., metabolic/genetic variants), or, factors specific to the stroke such as, (2) time since stroke, (3) the nature and location of the stroke, and finally (4) the level of motor impairment.

#### Individual Biological Variability

Intrinsic individual differences in neurotransmitter levels, metabolic factors, and genetic variants provide a complex source of variability when it comes to tDCS responsiveness. For instance, one study found a relationship between individual differences in baseline measures of GABA and anodal stimulation outcomes. However, this relationship was not found between GABA and cathodal or bi-hemispheric stimulation outcomes (46). Another study showed that individual differences in the level of dopamine receptor (D1) activity influenced the excitatory effects of anodal tDCS on M1 (47). These studies suggest that there is a complex, but influential, relationship between biological factors and stimulation response.

Another factor that has been widely explored is the role of an individual’s genetic variability on tDCS response. The secretion of brain-derived neurotrophic factor (BDNF), which is involved in the synaptic plasticity, neuronal growth, long-term potentiation (LTP) formation, and long-term memory storage (48–50), seems to be enhanced by the application of tDCS (51). Moreover, an individuals’ level of BDNF seems to influence the response to NIBS and especially to tDCS (28, 52). The most common form of BDNF polymorphisms, in which an amino acid substitution between a Valine and a Methionine at position 66 on the BDNF gene [Val66Met (53)], modulate the level of BDNF expression and are thus associated with a differential modulation of brain plasticity and altered motor plasticity (54). The Val66Met polymorphism, which induces less BDNF expression, has also been shown to be associated with less-efficient motor learning and reduced responsiveness to all forms of NIBS (28, 54, 55).

However, this finding is not always consistent. For example, one study did not find any impact of the BDNF polymorphism on the neuroplastic changes induced by anodal tDCS in older adults (56), while a second one failed to find any impact of the BDNF polymorphism on the neuroplastic-induced changes associated with either tDCS, transcranial random noise stimulation, or theta burst stimulation (57). Another study demonstrated that the BDNF polymorphism did not predict responses to tDCS in patients suffering from depression but that a serotonin transporter polymorphism did (58). This suggests that the impact of the BDNF polymorphism, or more globally, genetic polymorphisms on NIBS-related effects, may be disease or task dependent.

#### Time after Stroke

There are also a number of factors specific to individuals with stroke that may affect stimulation efficacy. One variable is that stimulation may be delivered at different times after stroke, during which different neural plasticity mechanisms (59, 60) are active, making it difficult to compare effects across time points. In addition, the categorical definitions of “time after stroke” may be variable as well. For instance, the “chronic phase” of stroke is defined across studies as patients anywhere from 3 months or more (up to many years) after stroke (61, 62). This means that within one “category” of patients with stroke, there may be enormous variability in the plasticity processes and thus the potential tDCS effects and interactions with those processes. More precise clarification of time after stroke, using absolute terms such as months, may be useful for comparing post-stroke tDCS results.

#### Nature and Location of the Stroke

The location of the stroke lesion (i.e., cortical or subcortical lesion) is similarly mixed or omitted in some tDCS studies (61, 63–66). However, neural plasticity processes and cortical reorganization inducing differential recruitment of different brain regions may be dependent of the size and location of the stroke (67). Another source of stroke-specific variability is the nature of the stroke (i.e., ischemic or hemorrhagic), with some studies mixing both populations together (18, 65, 66, 68). This could also be a potential confound as the deficits and brain plasticity processes associated with cortical/subcortical localization or ischemic or hemorrhagic stroke (69–71) could be different.

#### Level of Motor Impairment after Stroke

Finally, the level of impairment after stroke should be carefully considered. There is a wealth of literature that suggests that patterns of neural recovery may differ for individuals based on the severity of their stroke (72–76). The recruitment of different brain regions, such as the contralesional hemisphere, may also play a different role based on the level of motor impairment. Most of the tDCS studies in patients after stroke mixed patients with different level of impairments (from mild to severe) (18, 65, 77). However, it may be important to consider an individual’s level of motor impairment and optimal pattern of recovery when applying tDCS.

#### Interim Conclusion

These are just a few of the factors that may contribute to the variability of tDCS responsiveness in individuals after stroke. Such wide and complex factors present challenges to reproducibility in tDCS studies of stroke motor rehabilitation. In addition to the parameters mentioned here that may contribute to variability, it is also possible that other parameters that have not yet been explored may affect the efficacy of tDCS. For instance, fluctuating state-dependent interactions (78–81) with stimulation (e.g., attention, fatigue, mood, cognitive load) may be a source of intra-individual variability that is not well-studied or controlled for. Another factor is the design of the study, and particularly the use (or lack) of a sham control group. Studies that compare the effects of tDCS to another stimulation condition (e.g., anodal stimulation to cathodal stimulation), rather than to a sham or true baseline condition, may introduce further confounds when interpreting the effects of tDCS.

### Motor Paradigms during tDCS

In addition to sources of variability from the individual and stroke-specific factors, there are also many other experimental paradigm factors that could affect tDCS outcomes. While beyond the scope of the current review, there are many sources of noise that arise from the measurement of outcomes. For instance, the measurement of cortical excitability using TMS has been shown to be highly variable (82) and there is also noise within the measurement of performance on motor tasks (83, 84). In addition to measurement noise, there are also three additional sources of paradigm-related variability, specifically (1) the timing of the stimulation (e.g., before, during, or after training), (2) the motor task used, and (3) the emphasis on motor performance versus motor learning.

#### Time of Stimulation

Is the ideal time to apply tDCS before training to modulate the brain into an optimal state for learning, during training to reinforce training-induced plasticity, or after training to improve consolidation?

This is another question and potential source of variability when understanding the effects of tDCS on motor behavior. In healthy subjects, conflicting results show that tDCS can either be applied during motor training (85), or before training in order to increase corticospinal excitability (86). Other studies have examined stimulation after training to improve consolidation and retention (87, 88). However, the exact time during which tDCS can be applied to maximize neuroplasticity and evoke behavioral changes is still undetermined both in healthy individuals and in individuals with stroke. For instance, a single study with controversial results demonstrated that only tDCS applied before training was able to improve movement kinematics whereas tDCS applied during or after training induced degradation of the motor performance (36). However, other studies show that tDCS during training evokes similar or enhanced results compared to tDCS before motor training (63, 65, 66, 77, 89, 90).

Despite conflicting results in the literature, the most commonly used paradigm in stroke motor rehabilitation is to perform tDCS concomitantly with training on a motor task (see Table 1 for a list of studies that examined the effects of tDCS in individuals with stroke for upper limb motor recovery). Thus, while more research should be done in this area, another key question to consider is what type of task, and what parameters for the task should be implemented for optimal enhancement of function?

**Table 1:** **tDCS to enhance hand/arm motor function after stroke**.

PART 1				
Simple motor performance + tDCS				
*n*	Montage	Comparison group/results	Reference	
6	A and B over M1	Sham comparison group: improvement on JTT with both cathodal and anodal tDCS just after stimulation	(63)	

1	B over M1	Sham comparison group: improvement on JTT, pinch force, and simple reaction time just after stimulation	(64)	

6	B over M1	Sham comparison group: improvement on JTT immediately after stimulation and maintained 20 min after	(20)	

11	B over M1	Sham comparison group: improvement on both JTT and reaction time immediately after stimulation	(91)	

13	A and B over M1	Sham comparison group: both anodal and cathodal tDCS induce a significant reduction of reaction time	(65)	

19	C over M1	Sham comparison group: improvement on both digital dexterity (PPT) and the precision grip	(89)	

10	A, B, C and B with extracephalic reference electrode over M1	Sham comparison group: tDCS induces enhancement on JTT except when used with extracephalic reference electrode	(92)	

**Multiple sessions of tDCS combined with neurorehabilitation**				

*****n*****	**# of sessions (weeks)**	**Montage**	**Comparison group/results**	**Reference**

7	6	B over M1	No comparison group: improvement on JTT and upper limb function	(93)

9	5	A over M1	Sham comparison group: improvement on JTT maintained at 2 weeks after the end of the treatment	(94)

20	5	C over M1	Sham comparison group: improvement in both FMT and WMFT maintained 1 week after the end of the treatment	(61)

14	14	C over M1	Sham comparison group: improvement on JTT, FMT, and maximum grip strength maintained 4 weeks after the end of the stimulation	(18)

5	10	C over M1	No comparison group: improvement of motor function combined with an increased functional connectivity between M1 and dorsal premotor cortex (PMd) in the ipsilesional hemisphere	(62)

12	5	B over PMd	Sham comparison group: gains in motor function and dexterity accompanied by an increase in excitability of the contralesional rather than the ipsilesional hemisphere	(68)

18	5	A and B over M1	Between stimulation comparison (no sham): both anodal and cathodal tDCS induced motor function improvement	(95)

19	10	C over M1	Sham comparison group: tDCS had a role in motor imagery facilitation	(96)

20	20	B over M1	No stimulation comparison group: tDCS was more beneficial than functional training in order to improve motor function	(97)

40	6	A and B over M1	Sham comparison group: both anodal and cathodal tDCS enhanced rehabilitation induced motor function	(98)

59	15	A over M1	Cathodal stimulation alone and training alone comparison groups: cathodal tDCS combined with virtual reality therapy induced a greater improvement in motor function than each intervention alone	(99)

14	5	A over M1	Sham comparison group: cathodal tDCS combined with occupational therapy induced improvements in motor function	(100)

**Motor skill learning + tDCS**				

*****n*****	**Task**	**Montage**	**Comparison group/results**	**Reference**

12	SRTT	A over M1	Sham comparison group: cathodal tDCS improved motor skill learning compared to sham by 20%	(90)

18	VMT	C over M1	Sham comparison group: enhanced online motor skill learning and enhanced long-term retention (×10)	(66)

19	VMT	C over M1	Sham comparison group: enhanced online motor skill learning and enhanced long-term retention associated with functional brain reorganization	(77)

**PART 2**				

**Simple motor performance + tDCS**				

*****n*****	**Montage**	**Comparison group/results**	**Reference**	

12	A over M1	Sham comparison group: improvement of proximal upper limb motor function for mildly impaired patients but degradation for the more impaired patients	(101)	

12	B over M1	Sham comparison group: movement kinematics improved only with tDCS delivered prior training	(36)	

12	A, B, and C over M1	Sham comparison group: anodal and cathodal tDCS lead to superior motor performance improvements and changes in cortical excitability than bi-hemispheric tDCS	(46)	

9	A and B over M1	Sham comparison group: motor improvement is dependent on the shoulder abduction loading	(102)	

10	B over M1	Repetitive transcranial magnetic stimulation comparison group: in absence of combined motor practice, anodal tDCS failed to induce motor performance improvement	(103)	

16	B over M1	Sham comparison group: anodal tDCS induces motor performance improvements but failed to enhance the effects of 2 days in rehabilitation training	(104)	

**Multiple sessions of tDCS combined with neurorehabilitation**				

*****n*****	**# of sessions (weeks)**	**Montage**	**Comparison group/results**	**Reference**

18	10	A and B over M1	Sham comparison group: only cathodal tDCS (not anodal) is able to enhance rehabilitation induced motor function	(105)

23	10	C over M1	Sham comparison group: motor improvement only for patients with chronic and subcortical stroke	(37)

20	5	C over M1	Sham comparison group: bi-hemispheric tDCS reduces interhemispheric imbalance despite no observable clinical improvement	(38)

96	6	A and B over M1	Sham comparison group: neither anodal nor cathodal tDCS enhances bilateral arm training induced motor function	(106)

25	5	B over M1	Sham comparison group: anodal tDCS was not able to induce motor function improvement	(107)

20	15	B over M1	Sham comparison group: anodal tDCS was not able to induce a greater motor function improvement than sham but induced a reduction in wrist spasticity	(108)

*PART 1: studies that demonstrate a motor improvement with tDCS*.

*PART 2: studies that either do not demonstrate motor improvement with tDCS, or compare conditions and show conflicting results depending on the design*.

*tDCS, transcranial direct current stimulation; SRTT, serial reaction time task; VMT, visuomotor task; CIMT, constraint-induced movement therapy; JTT, Jebsen–Taylor test; FMT, Fugl–Meyer test; WMFT, Wolf motor function test; montage A, uni-hemispheric tDCS with cathode over contralesional hemisphere; montage B, uni-hemispheric tDCS with anode over ipsilesional hemisphere; montage C, bi-hemispheric tDCS*.

#### Motor Tasks

It seems that tDCS induces different outcomes depending on the nature of the motor task. For instance, one study showed that healthy subjects had different effects of tDCS depending on what task they performed. When they received tDCS during motor practice of a sequential finger tapping motor task, they showed online improvements. By contrast, when they received the same tDCS dose during motor practice of a visual isometric pinch force task, they only showed delayed, but not online, improvements (109). In individuals with stroke, tDCS applied during motor practice induces a strong and immediate improvement in dexterity of the fingers of the paretic hand (e.g., continuous improvement from the start of the tDCS application up until 30 min after the end of the stimulation). However, only a small delayed effect was reported on the kinematics of a paretic precision grip task (89). In addition, sensory feedback during motor performance and the bidirectional relationships within perceptual-motor tasks may also modulate the effects of tDCS on motor abilities after stroke (110, 111). These results suggest that the choice of and nature of a motor task is of crucial importance to maximize tDCS effects.

Depending on the goal or on the severity of the patients’ impairment, several tasks may be used concurrently with tDCS (Table 1). These may include simple motor tasks or more challenging tasks that engage motor, attentional and cognitive resources. The choice of task should be carefully considered in light of the variability it may introduce when used with tDCS. For more severely impaired patients who are not able to perform a motor task due to a lack of mobility or strength in the paretic hand/arm, several other paradigms have been developed to try to improve upper limb motor function. Motor imagery is one such task in which the mental representation of a movement (in individuals with stroke, a movement of the paretic upper limb) without any real/physical movement is used (112). tDCS has shown an improvement in the effects of motor imagery training in individuals with stroke (96). Another option is mirror therapy, in which a patient moves their unaffected limb and watches the movement in a mirror as though the moving limb were their affected limb. This is another alternative paradigm for more impaired patients, and, combined with tDCS, this paradigm has also demonstrated an ability to restore brain activation in the ipsilesional motor network and to enhance paretic upper limb motor function (113). Related to this, as well as the idea of perceptual-motor codes discussed previously, action observation therapy may also be useful for individuals after stroke by linking sensory and motor information to generate super-additive responses across the brain (110). These results suggest that tDCS can be an efficient add-on therapy even in the most impaired patients, although the choice of the motor task should be carefully considered.

### Motor Performance versus Motor Learning in Stroke

Finally, another major source of variability when evaluating the effects of tDCS on upper limb motor recovery after stroke is the outcome being measured. In particular, two terms that are often confused are motor skill learning and motor performance. They are often used interchangeably and not well delineated in the literature, introducing variability when understanding the effects of tDCS on stroke motor recovery. By definition, improvements in motor performance result in temporary improvement, whereas motor (skill) learning is a relatively permanent change leading to the acquisition of a new motor ability (114, 115). The effects of tDCS on motor learning versus motor performance are thought to involve different neural mechanisms. Immediate tDCS effects on brain activity/excitability, measured as motor performance, are thought to be mediated through the activation of sodium and calcium channels on the neuronal membrane (116). Polarity-dependent changes of cortical excitability after tDCS have already been documented (117). tDCS induces a depolarization or a hyperpolarization of the local membrane potential, thereby modulating the rate of action potentials and thus neuronal firing (27, 118–120), which are known to be associated with the modulation of motor performance in animals (121).

By contrast, the mechanisms associated with the effects of tDCS on long-term motor learning are thought to be mediated by the activation/insertion of glutamate, NMDA/AMPA, and γ-aminobutyric (GABA) receptors in the post-synaptic membrane (22, 116, 122, 123). These receptors mediate the induction of LTP and long-term depression (LTD) and are affected during tDCS and motor learning (124). The modulation of the glutamatergic system also leads to the synthesis of key proteins such as BDNF (28, 125), which is also involved in LTP formation (126, 127). Finally, there is also evidence that tDCS directly modulates LTP and LTD (128) and BDNF expression (51), which are the basic mechanisms involved in (motor) learning (50, 129–133).

Many researchers believe that motor learning, and especially motor skill learning, plays a key role in post-stroke recovery and neurorehabilitation (134–138). Thus, enhancement of motor skill learning is an attractive option for improving post-stroke motor recovery since it can lead to faster recovery, augment the effects of neurorehabilitation, and generalize to long-term, functional gains. Moreover, coupling tDCS and motor skill learning is likely to show long-term changes in stroke due to the similar molecular mechanisms between the two processes. For instance, both induce a change in membrane potential, increase the likelihood of neurons to fire, induce brain plasticity, and induce brain-derived neurotropic factor secretion (28, 139, 140). More plasticity during training leads to more learning and that leads to long-term gains. Currently, there are only three studies exploring the impact of tDCS on motor skill learning in individuals with stroke (Table 1). Two of them used bi-hemispheric tDCS over M1 during training on a visuomotor skill learning task and demonstrated an improvement of motor skill learning during the tDCS application. In addition, the improvement in motor skill was maintained 1 week later as shown in a delayed retention test (66, 77). The third study used cathodal tDCS over contralesional M1 during a serial reaction time task and demonstrated an improvement during a delayed retention test (90). More studies are of course needed to explore the effect of tDCS on motor skill learning specifically at the long-term retention level.

One reason for the small amount of studies exploring this topic is due to the interchangeable, but often incorrect, use of motor skill learning and simple motor performance in the literature, as well as the increased time and rigor required to carefully study long-term motor learning. However, there are more studies exploring the impact of tDCS on motor performance. These studies are split in two groups (see Table 1). The first group explored only immediate effects of tDCS on motor performance, typically in a single visit. The second group explored the repeated application of tDCS during daily neurorehabilitation therapy, such as occupational therapy, with the intent to enhance motor skill learning. However, oftentimes the “conventional therapy” is not in fact “conventional” and may be more or less rigorous than that which actually occurs in normal clinical practice. The use of this term without a careful description of what was actually done leads to a large amount of variability in the design of the therapy (intensity, regularity, amount of stimulation received) and the selected task [if a skilled movement is actually defined and trained (141, 142)]. Moreover, these studies did not specifically select a motor learning task and instead used general therapy, which could consist of diverse, goal-directed actions, or practice. This makes it difficult to measure actual changes in motor learning, as opposed to improvements in motor impairment or function. While these studies generally show positive effects that translate to real-world gains, it is difficult to say whether the tDCS was effective due to its role in driving motor learning, another mechanism for improvement. Given the extreme variability, it is not surprising that no clear and consistent impacts of tDCS have emerged.

## Electrode Placement

In addition to the aforementioned sources of variability, one of the key considerations in using tDCS to improve upper limb motor performance after stroke is where to stimulate the brain (e.g., neural target) and how (e.g., excitatory or inhibitory stimulation). The localization and polarity of the electrodes are especially critical in individuals with stroke due to the lesion location and potential spread of functional reorganization in the post-stroke brain (143). tDCS requires the use of at least two electrodes—one anode and one cathode. Under the anode, the resting membrane potential of the tissue depolarizes, leading to an increase in neuronal excitability. Under the cathode, the resting membrane potential of the tissue hyperpolarizes, leading to an inhibition of neural activity (27). In this next section, we will discuss the choice of the neural targets to improve motor behavior after stroke and the potential montage options.

### Neural Targets

Neuroimaging studies have identified sensorimotor and premotor areas as key targets associated with functional motor recovery after stroke (17, 144, 145). NIBS allows researchers to take these neuroimaging findings and test the causal relationship between identified regions and functional recovery by stimulating the regions and observing the effects. In this section, we discuss key neural targets for tDCS to enhance stroke motor recovery.

#### Primary Motor Cortex

The primary motor cortex (M1) is located in the dorsal portion of the frontal lobe along the precentral gyrus. It is one of the principal brain areas involved in motor function and works to plan and execute movements (146, 147). Signals from M1 cross the body’s midline to activate the opposite side of the body (i.e., the left hemisphere controls movements on the right side of the body). Every part of the body is represented in M1, and these representations are arranged somatotopically. The amount of brain matter dedicated to any body part is related to the amount of control that M1 has over that body part (148). Motor neurons that originate in the motor cortex aggregate in fibers that travel from the cerebral cortex to the brain stem. This tract is named the corticospinal tract. A stroke that affects the corticospinal tract can induce motor impairments. The following sections describe why M1 has been and still is a neural target of choice in order to improve motor recovery after stroke.

In healthy individuals, each hemisphere provides reciprocal inhibitory connections to the other, which are thought to promote coordination between the two hemispheres (19, 149). However, after a stroke, these normal inhibitory connections can become abnormal and lead to greater dysfunction. The interhemispheric rivalry hypothesis suggests that the contralesional M1 may exert an abnormal increase in interhemispheric inhibition of the ipsilesional M1 (3, 14, 15, 150), inducing an additional dysfunction in the ipsilesional hemisphere. Motor impairments may thus arise from the lesion itself as well as from the additional abnormal inhibition patterns coming from the contralesional hemisphere. This can lead to a decrease in excitatory output from the ipsilesional M1 and interfere with functional motor recovery on the affected side. Accordingly, excitation of ipsilesional M1 and/or inhibition of contralesional M1 may be viable options in stroke studies to enhance motor function (151).

Since Priori and collaborators in 1998 described the tDCS as a potential way to modify cortical excitability (26), the primary motor cortex (M1) has become a choice target for most stroke tDCS paradigms. It is worth noting that the emphasis on M1, even in healthy volunteers, may also reflect a bias due to the ease of targeting M1. Stimulation of M1 by TMS can evoke a measurable, and visually observable, movement in the contralateral hand or arm. Typically, this effect is measured using surface electromyography in the contralateral hand and revealed as a motor-evoked potential (MEP). Stimulation over M1 allows for the measurement of amplitude and width of the MEP as a measure of changes in cortical excitability, with an increased MEP amplitude typically indicative of increased cortical excitability. M1 is thus an ideal target as it allows for both easy localization of the target area and quantifiable, measurable effects of stimulation. For other areas, alternative approaches like localization by the EEG 10-20 system (152–157) or by neuronavigation systems (158) must be used, and effects of stimulation are typically measured by performance on various behavioral tasks.

As M1 is a common target for enhancing post-stroke motor function, and specifically behavioral changes, numerous studies have examined this paradigm. Many studies have shown positive results in enhancing motor function in patients with a large range of motor deficits using tDCS (77, 90, 159). Despite positive findings, other recent studies have shown negative or null findings with M1 stimulation, revealing the high variability of observed effects of tDCS over M1 stimulation (37, 46, 102, 106). These studies suggest that some inter-individual variability could influence the response of tDCS stimulation over M1. These conflicting results (see Table 1) also suggest that a one-size-fits-all method is not the best approach for tDCS in stroke recovery, and that potentially adapting the choice of the target area, among other parameters, to each individual could lead to better gains.

#### Dorsal Premotor Cortex (PMd)

The PMd is part of the premotor regions and is involved in movement selection (160–163), another well-known area in the field of motor recovery. In a middle cerebral artery stroke, major portions of the primary motor and sensory cortices can be damaged, leading to motor impairments. Due to the distribution of the vasculature, PMd is often spared and is one of the remote motor areas recruited in the ipsilesional hemisphere during movements with the paretic hand after a stroke (164–166). PMd recruitment is thus thought to be a compensatory response of the brain, which supports enhanced paretic hand function (166–169). Recent studies suggest that successful motor skill learning is strongly correlated with PMd recruitment in individuals with stroke (77, 170). Accordingly, PMd is a strong tDCS target candidate for enhancing post-stroke motor recovery (145). Some studies using rTMS demonstrated an enhancement of motor function in individuals with stroke either when targeting ipsilesional (excitation) (171) or contralesional (inhibition) (172) PMd, and recently, one study confirmed the positive impact of stimulating the premotor area with tDCS in enhancing motor function after stroke (68).

The small number of studies using PMd as neural target in order to improve motor function might be explained by the assumption that given the large size of tDCS electrodes and the current spread (173), PMd could also have been peripherally stimulated during tDCS with electrodes centered over M1. The use of HD-tDCS and more focal stimulation paradigms may provide a way to disentangle these contributions.

#### Supplementary Motor Area (SMA)

The SMA is medial to the premotor cortex and is involved in movement control, bilateral movement coordination, and sequential motor learning (174–176). Similarly to PMd, the SMA is a region that is known to be involved with the post-stroke compensatory motor network (177, 178). Moreover, it seems that SMA recruitment is highly associated with an increased recovery of motor function (i.e., individuals with good recovery show reductions in task-evoked activation in the SMA over time, which is associated with an improvement in motor function) (179). This pattern of increased recruitment during the early stages of learning, and a reduction of this recruitment when the task has been consistently learned (180), is seen both in individuals with stroke and in healthy individuals when they learn a new motor skill. Promising results from studies on healthy individuals suggest that excitatory NIBS [both rTMS (181) and tDCS (182, 183)] over the SMA is associated with an improvement of motor skill learning. To date, there are no published studies exploring the impact of tDCS directly over the SMA to enhance motor function after stroke. However, the SMA was thought to also have been stimulated during PMd stimulation (68), which showed gains in motor function and dexterity of the paretic arm from excitatory stimulation over PMd/SMA. Moreover, in healthy individuals, SMA activation seems highly correlated with successful motor skill learning suggesting a key role of the SMA in the correct acquisition of a new motor skill (184). Since the more similar the reconfigured network is to the original undamaged network, the better the recovery (17, 185, 186), stimulating areas that are spontaneously involved during motor skill learning in healthy individuals such as the SMA could be an effective option for enhancing motor recovery.

#### Cerebellum

Historically, the cerebellum has been described as a part of the motor system because cerebellum lesions can lead to errors in movement control or coordination (187). A recent study also demonstrated the key role of the cerebellum and especially of lobules V and VI in sequential motor skill learning (188). Several observations have provided evidence that the cerebellum is part of the motor recovery network after stroke as well. At the metabolic level, immediately after a stroke, blood flow in the cerebellar hemisphere contralateral to the lesion (i.e., ipsilateral to the paretic arm) decreases, even if there is no lesion in the cerebellum (189). Interestingly, as the patient regains motor abilities in the first few weeks after the stroke, blood flow in the cerebellum likewise increases (190). That is, the rate of increase in blood flow in the contralesional cerebellum correlates with the rate of motor recovery in the patient, suggesting a relationship between the two. Moreover, at a functional level, the intensity of cerebellar recruitment during a motor task performed with the paretic hand is correlated with motor recovery. After several months, there is a reduction of the recruitment in this area during task practice of the affected hand, suggesting a transient use of spared neural resources in the cerebellum during the recovery process (191). Nevertheless, it has to be noted that these results suggest only a correlation between cerebellum function and motor recovery and not a causal relationship.

Recently, the ability of tDCS to modify cerebellar excitability has been demonstrated in combined tDCS–TMS experiments (192). In a visuomotor transformation paradigm, anodal tDCS over the cerebellum was shown to enhance adaptation learning (193, 194). While tDCS over the cerebellum seems to enhance post-stroke cognitive functions (195), as yet there have been no studies demonstrating that modifying cerebellar excitability with tDCS also improves post-stroke motor function.

#### Other Areas

In addition to these areas, several other regions of the brain could be promising targets as well. The primary and secondary motor areas have strong connections with the parietal cortex, and it may be possible to perturb the motor network through stimulation over the parietal cortex. The fronto-parietal network is largely associated with deficits in motor planning (e.g., apraxia) (196–198), and the parietal cortex is also involved in motor skill learning (199) in individuals with stroke (170). In addition, tDCS over the parietal cortex has been shown to improve hemi-neglect in individuals with stroke, which may have tangential effects on motor recovery (200). However, currently no studies have used this montage to examine post-stroke motor recovery.

The DLPFC is another region that plays an important role in working memory and may be a key component of the motor learning network both in healthy individuals (201) and individuals with stroke (202), especially for complex tasks in which there is a cognitive component (203). More studies are needed to explore the ability of tDCS on DLPFC to enhance post-stroke motor recovery.

Finally, some studies have also demonstrated an involvement of the primary somatosensory cortex (S1) in post-stroke motor recovery (204, 205). tDCS over S1 leads to variable improvements in healthy subjects and in individuals with stroke on performance in the somatosensory domain. For instance, bi-hemispheric tDCS enhanced post-stroke tactile discrimination in individuals after stroke and anodal tDCS enhanced tactile discrimination in healthy subjects. However, no effects were observed after cathodal tDCS in healthy subjects (206–208). Previous studies have shown improvements in motor learning associated with excitatory rTMS over ipsilesional S1 (209) or with continuous theta burst stimulation over the contralesional S1 (210). However, again there have not been any published studies exploring the use of tDCS directly over S1 to enhance post-stroke motor function, although it is likely also stimulated during M1 stimulation with larger electrodes. More targeted stimulation of only S1 thus represents an interesting option for future research.

Overall, it seems that while studies exploring tDCS for motor recovery are growing exponentially, they have been mainly limited in their neuroanatomical targets primarily to M1 [except for one study targeting PMd/SMA (68)]. It is possible that the lack of published studies in stroke recovery on the use of tDCS over regions besides M1 is biased due to a potential lack of any positive, significant results. If this is the case, it would be beneficial for the field of stroke recovery to encourage the publication of all tDCS studies, even the ones with no significant or negative results, to at least understand what has been tried. It is likely that stimulation over different motor regions with tDCS may lead to specific gains in specific post-stroke behavioral conditions with specific populations. In this regard, the field would benefit from better understanding where it does, and does not, work.

### Monocephalic or Bi-Hemispheric Montage

In a conventional tDCS setup, tDCS is applied using two electrodes (one anode and one cathode). The position of the electrodes is typically driven by the goal of the study. Depending on this goal, tDCS can be applied in two distinct montages (monocephalic/bi-hemispheric). Nevertheless, the applied current is not restricted over the targeted area but is mode widely distributed with a hotspot around the active electrode (see next paragraph) (173).

When tDCS is applied to change excitability by targeting a single area, this is considered a monocephalic montage. In this case, one small electrode (the active electrode) (up to 50 cm^2^) is placed over the desired neural target (i.e., the brain area in which the excitability changes induced by the stimulation is thought to be the more useful regarding the goal of the study) and a larger neutral electrode called the “return electrode” (up to 100 cm^2^) is placed over the contra-orbital area. Increasing the size of the electrode (and keeping its current strength constant over time) will reduce the overall current density flowing through that electrode and thus eliminate its functional efficacy (211). To restore excitability in the ipsilesional hemisphere or rebalance interhemispheric interactions in order to enhance motor function, two different monocephalic montages are typically used: (1) upregulating the excitability of the ipsilesional hemisphere using a monocephalic montage with the anode as the active electrode over the ipsilesional hemisphere and the cathode as the return electrode (20, 91) and (2) downregulating the excitability of the contralesional hemisphere, using a monocephalic montage with the cathode as the active electrode over the contralesional hemisphere and the anode as return electrode (63, 101, 212).

tDCS can also be applied in a bi-hemispheric manner, permitting simultaneous coupling of excitatory and inhibitory effects. The bi-hemispheric montage uses two electrodes of the same size in order to modify brain excitability by targeting two neural targets. Notably, one electrode should be the anode and the other the cathode. With this montage, the effects of both monocephalic montages previously mentioned are coupled with the anode over the ipsilesional hemisphere and the cathode over the contralesional hemisphere (18, 61, 89). The effect of the bi-hemispheric montage is supposed to be linked to a modulation of interhemispheric inhibition. While interhemispheric inhibition as a result of the bi-hemispheric montage has been directly demonstrated in healthy subjects (213), to our knowledge, such a direct demonstration has not yet been performed in stroke patients. In healthy individuals, several direct comparisons have demonstrated that the use of bi-hemispheric tDCS drives at least as much improvement of motor performance as using a monocephalic montage (214, 215) and induces a stronger modulation of the connectivity between the two M1 regions compared to the monocephalic montage (216, 217). However, a single study in individuals with stroke did not demonstrate improved efficiency of bi-hemispheric tDCS in increasing post-stroke motor function compared to a monocephalic montage (46). Furthermore, a deterioration of motor function in the paretic upper limb has been observed after monocephalic cathodal tDCS (101, 218) over the contralesional hemisphere in more impaired patients whereas an improvement is observed in the less impaired patients, suggesting that the optimal parameters may depend on the severity of the stroke. This may, again, potentially explain some of the variability in tDCS stroke findings.

Finally, HD-tCDS refers to the use of more than two electrodes, usually smaller in size, in order to distribute the positive and negative current flow to target neural regions with greater spatial resolution. This is discussed further in Section “Focal versus Broad Stimulation” below.

To date, there is little clear evidence regarding the best tDCS montage to improve post-stroke motor function. Once again, the same montage may likely not work for all patients and might be tailored based on lesion location, severity of motor impairment, desired outcomes, or other factors.

### Return Electrode Positioning

A related issue is the placement of the return electrode for the “monocephalic” montages. tDCS involves the flow of current through the brain, which means that there must always be a positive pole and a negative pole. However, when one wishes to only induce a positive or negative effect across the brain, the second electrode is often referred to as the “return” electrode. However, the return electrode can still evoke an effect on the tissue underneath. The primary way to avoid an effect of the return electrode is to use a larger electrode, as the larger an electrode is, the more inactive/passive it will be, since it will reduce current density induced by it (211). Many studies incorrectly mention the use of a control/return electrode when in reality, studies that use 35 cm^2^ electrodes (or smaller) as return electrodes are implementing bi-hemispheric tDCS and inducing a modulation over the brain area for this “return” electrode. Unfortunately, the primary way to avoid this effect is to use a large return electrode, which is not always easy and/or feasible. However, not doing this introduces the risk of an undesirable current from the return electrode.

To circumvent this problem, one option is to use an extracephalic return electrode (e.g., on the shoulder or neck). In the past, this option has been excluded to avoid stimulation of the brainstem due to safety concerns that arose from early studies (219, 220). Recently, however, several studies have demonstrated safety using extracephalic montages (221, 222). To date, only a single study testing the impact of tDCS on motor performance using an extracephalic electrode has been implemented (92). There is also one study testing the impact of tDCS with an extracephalic electrode on motor skill learning (223). While in this study, the use of tDCS did not improve motor function, future studies should continue to explore both the feasibility and the potential benefits of using an extracephalic return electrode for tDCS in the stroke population.

### Focal versus Broad Stimulation

One point that is often considered a major drawback in using tDCS is the low spatial resolution of the induced current (211). In contrast to rTMS, which provides a more focal stimulation on the order of millimeters (224), the current flow delivered by conventional tDCS is not limited to the area under the electrodes for several reasons: (1) the size of the used electrodes (15–50 cm^2^) is oftentimes larger than the target region of interest and more likely spread to adjacent cortical areas and (2) as previously mentioned, the tDCS induced-current flow is not limited to the neural target but widely distributed contiguously between the two electrodes.

High-definition tDCS is appealing option to use tDCS in a more focal way. HD-tDCS also leads to an increase in cortical excitability, as measured by an increased MEP amplitude up to 2 h after the stimulation, compared to conventional tDCS (30). While the impact of HD-tDCS has shown an improvement of the naming accuracy on aphasia in patients with stroke (225), there are not yet any published studies showing the impact of HD-tDCS on motor function in individuals with stroke.

However, in stroke rehabilitation, the spread of current to affect many regions within a network may in fact be beneficial. While more research is needed to compare focal to broad stimulation in individuals after stroke, the effects of conventional (broad) stimulation in stroke recovery have been generally positive. The conventional tDCS setup modulates functional connectivity (FC) and regions distal to the stimulated region (226, 227) [for a review, see Liew and colleagues (35)], which is important as FC is often disrupted in individuals with stroke (4). Several studies show interesting results of connectivity changes following tDCS in stroke: (1) bi-hemispheric tDCS over Broca’s area (anode over the left hemisphere) increased FC in the left hemisphere after treatment for 3 weeks, (2) 10 sessions of conventional bi-hemispheric tDCS over M1 combined with occupational therapy induced an improvement of the FC between ipsilesional M1 and contralesional premotor cortex (62), and (3) bi-hemispheric tDCS over M1 applied in a single session combined with training on a motor skill learning task induced a reorganization of FC in the motor network with a specific improvement in FC between PMd and M1 in the ipsilesional hemisphere (228). An appealing option, made possible by HD-tDCS, could be to stimulate not only a part of the network (such as M1), but to try to specifically stimulate the entire motor network in order to enhance post-stroke motor network connectivity and subsequently motor function.

From a neurophysiological point of view, focal stimulation is thought to be superior as it allows researchers to directly target the region of interest with less stimulation on other regions. However, it is feasible that, from a neurorehabilitation point of view, concomitant stimulation of adjacent cortical areas of the motor network by conventional tDCS with larger electrodes is beneficial (135, 210). Further research is needed in these areas.

### Lesioned Regions

Finally, according to modeling studies, when applying tDCS, the current flow is dependent on the electrode montage (i.e., the position and size of electrodes), the conduction of the different tissues (e.g., skin, skull, white and gray matter, cerebrospinal fluid, etc.), and on individual variation in anatomical features (229–231). Thus, an important question to address when using tDCS in participants after stroke is whether the lesion itself may perturb the current flow. This question is important since it is possible that the stroke lesions induce perturbations during stimulation, as found with rTMS over the post-stroke brain (232). Studies exploring the influence of anisotropic conductivity in the skull and white matter have demonstrated that the spatial distribution of current density is altered by changes in white matter anisotropy (233, 234) and also by variations in the gyri and sulci of each brain (235). This suggests that inter-individual variation in brain structure and integrity need to be taken into account when positioning electrodes. However, in individuals with stroke, it seems that even though there is a perturbation due to the lesion (with an increase in current density at the lesion borders), the global current density remains relatively unchanged compared to the healthy brain (236). Yet again, additional studies are needed to better understand and model current flow in the post-stroke brain.

### Summary

As demonstrated in this review, the field of investigation remains wide and varied, and the path to find an efficient and well-defined therapeutic tool is still far ahead. It is possible that one of the neural targets described above may drive stronger and more reliable effects, although it is more likely that there are complex interactions between many of these stimulation parameters, including neural target, task choice, and timing and duration of stimulation. Additional studies are required to determine the best tDCS target region in order to improve post-stroke motor function and the best way to stimulate its function. On the other hand, targeting any of the areas inside the motor learning network may drive the same enhancement effect through their connections to one another. The regions noted here may all be entryways into modulation of the motor network, which are likely to induce both changes specific to each stimulated site as well as similar global changes regardless of the site. Moreover, the one-size-fits-all approach used in most studies may not be an optimal approach. As recently suggested (237), an individual neural target selection based on the functional recruitment during a specific behavior may better permit enhancement in each individual. In other words, at the individual level, the key area involved in a specific task could be selected as a neural target for tDCS application. To do this, the precise and individually based selection of the neural target using a neuronavigation system, as often used with TMS studies, would be beneficial.

## Conclusion

In recent years, there has been an increase in the number of studies using tDCS to enhance post-stroke motor recovery. Despite the positive results observed in many studies, there is large variability and a lack of reliability in the literature. The optimal parameters, such as the neural target, the nature of the stimulation, the timing of stimulation in relation to a task, and more, need further exploration before tDCS can readily be used as a therapeutic tool in clinical settings. Moreover as suggested by Grefkes and Fink (238), large, randomized, multicenter controlled trials are now needed to determine the optimal design to enhance motor function after stroke using tDCS. The present review does not question the ability of tDCS to modify brain excitability, restore interhemispheric interaction, and enhance motor learning but rather suggests that to best promote neurorehabilitation processes, a better understanding of how we can maximize each parameter of use is needed.

## Author Contributions

SL: performed the review and drafted the manuscript. S-LL: performed the review and drafted the manuscript. All authors edited and revised the manuscript.

## Conflict of Interest Statement

The authors declare that the research was conducted in the absence of any commercial or financial relationships that could be construed as a potential conflict of interest.
